# Significant Quantitative Differences in Orexin Neuronal Activation After Pain Assessments in an Animal Model of Sickle Cell Disease

**DOI:** 10.3389/fmolb.2020.00005

**Published:** 2020-01-31

**Authors:** Kimberlei Richardson, Nia Sweatt, Huy Tran, Victor Apprey, Subramaniam Uthayathas, Robert Taylor, Kalpna Gupta

**Affiliations:** ^1^Department of Pharmacology, Howard University College of Medicine, Washington, DC, United States; ^2^Division of Hematology, Oncology and Transplantation, Department of Medicine, University of Minnesota Medical School, Minneapolis, MN, United States; ^3^Department of Family Medicine, Howard University College of Medicine, Washington, DC, United States; ^4^Division of Hematology/Oncology, Department of Medicine, University of California-Irvine School of Medicine, Irvine, CA, United States

**Keywords:** orexin, hyperalgesia, sickle cell, pain, hypocretin

## Abstract

Sickle cell disease is a hemoglobinopathy that causes sickling of red blood cells, resulting in vessel blockage, stroke, anemia, inflammation, and extreme pain. The development and treatment of pain, in particular, neuropathic pain in sickle cell disease patients is poorly understood and impedes our progress toward the development of novel therapies to treat pain associated with sickle cell disease. The orexin/hypocretin system offers a novel approach to treat chronic pain and hyperalgesia. These neuropeptides are synthesized in three regions: perifornical area (PFA), lateral hypothalamus (LH), and dorsomedial hypothalamus (DMH). Data suggest that orexin–A neuropeptide has an analgesic effect on inflammatory pain and may affect mechanisms underlying the maintenance of neuropathic pain. The purpose of this study was to determine whether there are neuronal activation differences in the orexin system as a result of neuropathic pain testing in a mouse model of sickle cell disease. Female transgenic sickle mice that express exclusively (99%) human sickle hemoglobin (HbSS-BERK) and age-/gender-matched controls (HbAA-BERK mice; *n* = 10/group, 20–30 g) expressing normal human hemoglobin A were habituated to each test protocol and environment before collecting baseline measurements and testing. Four measures were used to assess pain-related behaviors: thermal/heat hyperalgesia, cold hyperalgesia, mechanical hyperalgesia, and deep-tissue hyperalgesia. Hypothalamic brain sections from HbAA-BERK and HbSS-BERK mice were processed to visualize orexin and c-Fos immunoreactivity and quantified. The percentage of double labeled neurons in the PFA was significantly higher than the percentage of double labeled neurons in the LH orexin field of HbAA-BERK mice (^*^p < 0.05). The percentages of double labeled neurons in PFA and DMH orexin fields are significantly higher than those neurons in the LH of HbSS-BERK mice (^*^p < 0.05). These data suggest that DMH orexin neurons were preferentially recruited during neuropathic pain testing and a more diverse distribution of orexin neurons may be required to produce analgesia in response to pain in the HbSS-BERK mice. Identifying specific orexin neuronal populations that are integral in neuropathic pain processing will allow us to elucidate mechanisms that provide a more selective, targeted approach in treating of neuropathic pain in sickle cell disease.

## Introduction

Sickle cell disease (SCD) is characterized as a hemoglobinopathy that causes red blood cells to sickle, and pain experienced by those individuals who suffer with SCD is associated with significant morbidity and increased death. In the United States, SCD accounts for over $450 million in healthcare costs each year (Steiner and Miller, 2006; Hassell, 2010) and there is a lack of knowledge related to the development and treatment of neuropathic pain associated with SCD. According to the International Association for the Study of Pain, neuropathic pain is defined as “pain arising as a direct consequence of a lesion or disease affecting the somatosensory system either at the peripheral or central level” (Haanpaa et al., 2011; Molokie et al., 2011). It is possible that altered processing within the nervous system may be the cause for persistent and sometimes unrelieved neuropathic pain in SCD.

Neuropathic pain has not been well-studied in patients with SCD to date. It is estimated that the incidence of neuropathic pain in the SCD population may be twice as what is found in other chronic pain populations other than SCD (Brandow et al., 2014). The defining characteristics of neuropathic pain are allodynia and hyperalgesia (Ballas and Darbari, 2013). Classical components of neuropathic pain are pain from a non-painful stimulus (i.e., extreme sensitivity to cool stimuli) and increased pain from a painful stimulus and pain caused by a stimulus that what would normally not be characterized as painful (Treede et al., 1992; Sethna et al., 2007).

Optimal management of neuropathic pain is yet to be delineated and opioid and non-steroidal anti-inflammatory drugs (NSAIDs) have not provided treatments that effectively alleviate neuropathic pain. While this improvement in treatment options for neuropathic pain research have been observed, pain is not always properly managed (Brandow et al., 2014). In order to develop better treatment strategies, it is important to identify neurochemical processes that may be involved in mediating neuropathic pain and use this info to develop better treatment regimens. One possible system to explore is the orexin system since it has been reported to mediate pain. The orexin system offers a novel approach to treat chronic pain and hyperalgesia. This system has been linked to the mediation of neuropathic pain and inflammatory processes (Yamamoto et al., 2002; Razavi and Hosseinzadeh, 2017); however, no published studies have investigated its possible role in SCD.

This current study utilizes transgenic sickle mice that express human sickle hemoglobin (HbSS) to explore the possibility of the orexin system as a target region in mediating neuropathic pain in SCD. Orexins are a family of hypothalamic peptides that play a role in the regulation of feeding behavior, energy metabolism, reward, and the sleep-wake cycle (de Lecea et al., 1998; Sakurai et al., 1998; Aston-Jones et al., 2009; de Lecea, 2012). Orexin neurons are expressed in the dorsomedial hypothalamus (DMH), perifornical area (PFA), and lateral hypothalamus and send their projections into other brain regions (Peyron et al., 1998, Chen et al., 1999; Nambu et al., 1999). Some of these regions are involved in analgesia and play a role in descending pain inhibition (Ossipov et al., 2010). There are two orexinergic receptors and orexin 1 receptor has a greater affinity for orexin A vs. orexin B peptide (Trivedi et al., 1998; Lu et al., 2000; Marcus et al., 2001). It has been demonstrated that orexinergic projections from the hypothalamus project to the spinal cord (lamina I) (van den Pol, 1999), lamina X, and laminae II–VII in the dorsal horn (Date et al., 2000; Bingham et al., 2001). Data suggest that orexin-A has an analgesic effect on inflammatory pain (Yamamoto et al., 2002), but it is not clear if the same mechanisms underly the maintenance of neuropathic pain and inflammatory pain. It is also not known whether the same analgesic effect with orexin-A on inflammatory pain will be similar in a neuropathic pain model. Neuropathic pain can be difficult to manage with standard analgesics such as opioids (Arner and Meyerson, 1988). Hence, the orexin system may offer a novel approach to treat chronic pain and hyperalgesia.

Enhanced pain-related behaviors have been observed in adult mice after temporally-controlled ablation of orexin neurons (Inutsuka et al., 2016). The mechanism by which orexin system modulate neuropathic pain is not well-established in the literature. Before it can be determined how the orexin system is involved in the mediation of neuropathic pain in a model of SCD, it is important to determine whether factors associated with neuropathic pain (i.e., hyperalgesia) differentially influence orexin neuronal activity. Therefore, the purpose of this study was to identify whether there were activational and topographical changes in the various subpopulations of orexin neurons as a result of various pain assessments in a mouse model of neuropathic pain in sickle cell disease. Identifying and understanding the activity of this neuronal circuitry will allow us to gain better perspective on differential patterns of activity in orexin neurons in the DMH, PFA, and LH after pain testing. The data from these experiments can lay the foundation for a more in-depth investigation on alternative pharmacological therapies to treat neuropathic pain in the SCD population by directly targeting the subpopulations that can influence nociceptive processing and reduce hyperalgesia. These studies can move the field forward by identifying whether there are selective subpopulations of orexin neurons that may be preferentially recruited during neuropathic pain. In this study, we established baseline measurements for pain responses and assessed orexin neuronal activation in the DMH, PFA, and LH of transgenic mice expressing human sickle hemoglobin (HbSS-BERK) and control mice expressing normal human hemoglobin A (HbAA-BERK).

## Materials and Methods

### Animals

Female transgenic HbSS-BERK sickle mice and age-/gender-matched controls (HbAA-BERK) were used in this study (*n* = 10/group, ~4–6 months old, 20–30 g). The HbSS-BERK express human (99%) sickle hemoglobin and HbAA-BERK control mice express normal human hemoglobin A (HbAA). Females more commonly express neuropathic pain in pain populations, including SCD (Torrance et al., 2006; Butler et al., 2013; Brandow et al., 2014). The mice were bred and characterized by phenotype in a pathogen-free facility under a 12 h light-dark cycle at the University of Minnesota. The HbSS-BERK mice display similar pathological features of human SCD such as hematologic disease, organ damage and tonic hyperalgesia (Paszty et al., 1997; Kohli et al., 2010; Giuseppe Cataldo et al., 2015). All animal care and experimental procedures were reviewed and approved by the Institutional Animal Care and Use Committee at the University of Minnesota.

### Behavioral Assessments

All behavioral tests were performed in a quiet room at a constant temperature (23–25°C). All mice were habituated to each test protocol and environment. Before performing baseline measurements and testing, four parameters were used to assess behaviors in the following order of testing: mechanical hyperalgesia, thermal hyperalgesia, grip force, and cold hyperalgesia (Kohli et al., 2010).

### Mechanical Hyperalgesia

To assess mechanical hyperalgesia, each mouse was put on a wire mesh apparatus under a glass container, allowed to acclimate, and a von Frey filament was applied to the hind paw for 1–2 s. A 1.0 g (4.08 mN) von Frey (Semmes-Weinstein) monofilament (Stoelting) was applied to the plantar surface of the hind paw of each mouse with enough force to bend the filament. Paw withdrawal frequency was determined by the number of time paw lifting was observed per 10 applications.

### Thermal/Heat Hyperalgesia

Thermal hyperalgesia was determined via measurement of heat sensitivity in the HbAA-BERK- and HbSS-BERK mice. Thermal hyperalgesia was assessed using the Hargreave's apparatus with a radiant heat stimulus. As previously described (Kohli et al., 2010; Lei et al., 2016; Tran et al., 2019), a radiant heat stimulus was applied under the hind paws of each mouse following acclimation to the floor of the Hargreave's apparatus. The radiant heat stimulus was located under the glass floor and administered using an infrared heat source. The paw withdrawal latency was recorded as the time when the mouse withdraws its paw from the heat stimulus (to the nearest 0.1 s).

### Grip Force

To assess deep tissue hyperalgesia, a digital grip force meter (Chatillon) was used to measure peak forepaw grip force. The force was measured by gently holding each mouse by its tail and pulling it across a wire mesh gauge. The grip force was recorded as the force (in g) exerted at the time of grip release by each mouse.

### Cold Hyperalgesia

Cold hyperalgesia was determined via measurement of cold sensitivity of the mice to a cold plate set at 4°C. Cold withdrawal latency was determined by the time it took each mouse to initially lift either forepaw. Cold withdrawal frequency was determined by the number of times that mouse lifted and rubbed the forepaws over a period of 2 min.

### Immunohistochemical Processing for c-Fos and Orexin in Hypothalamic Sections

In preparation for double label immunohistochemistry, brains from each mouse were extracted 90 min after behavioral testings and immersed in 10% formalin for fixation for at 1–2 weeks. Following cryoprotection in 30% sucrose solution, coronal brain sections were cut and processed for c-Fos and orexin-A as previously described (Richardson and Aston-Jones, 2012). Sections were incubated overnight at room temperature in primary antibody against Fos-related antigens (1:1,500, SC-52, Santa Cruz), then rinsed and incubated for 2 h with secondary antibody (biotinylated donkey anti-rabbit 1:500, Jackson Immunoresearch Laboratories). Sections were transferred to avidin–biotin complex (ABC, 1:500, Vector Laboratories) for 1.5 h and then Fos neurons were visualized by placing the sections in SIGMAFAST 3,3′-diaminobenzidine (DAB, D8552, Sigma) with cobalt chloride metal enhancer. Following a 45 min incubation in PBS-azide, the sections were placed in primary antibody for orexin-A (1:1,000, SC8070, Santa Cruz) overnight. Sections were incubated in secondary antibody (biotinylated donkey anti-goat 1:500, Jackson Immunoresearch) the next day, incubated in ABC and then orexin neurons are visualized using DAB (D5637, Sigma, no metal enhancer) with 0.0002% H_2_O_2_. The sections were dehydrated through graded alcohols, cleared in xylene, and coverslipped with Permount. Orexin-positive neurons exhibited brown cytoplasmic staining and Fos-positive nuclei (cobalt chloride intensified) were stained black.

### Quantification of Neurons and Statistical Analysis

The number of neurons with Fos positive nuclei, orexin-A positive cytoplasmic staining, and double labeled Orexin-Fos neurons was counted in the DMH, PFA, and LH for the HbAA-BERK and HbSS-BERK mice. The area located medial to the fornix was defined as the DMH region, the region located around the fornix was defined as the PFA region and the region lateral to the fornix was defined as the LH region (similar to other studies (Harris et al., 2005; Richardson and Aston-Jones, 2012). Quantification of the labeled neurons was conducted using a unique number code for each animal so that the investigator was blinded to the treatment groups.

Hypothalamic sections at two different levels, rostral (Bregma −1.34 mm) and caudal (Bregma −1.94 mm) (Paxinos and Franklin, 2001) level from each animal were used to count orexin- and Fos- positive neurons. A representative section from the rostral and caudal orexin fields of each animal was used to ensure that there was a good representation of the hypothalamic field, as described in Richardson and Aston-Jones (2012). A color image of the orexin field was acquired from a digital camera at 10× -20× magnification using brightfield illumination from a light microscope (Zeiss) connected to a computer station that capture images. The labeled neurons were marked using a pointer tool in Zen Pro software, preventing a cell from being counted more than once in an image. Neurons were counted bilaterally for each region and at each level (Zen Pro software [Carl Zeiss Microscopy, LLC, White Plains, NY]). The data are expressed as average counts of Fos positive, orexin positive, and percentage of double-labeled neurons (total number of double labeled neurons divided by total number of orexin positive neurons).

### Statistical Analysis

Behavioral assessments and neuron counts for each hypothalamic region were quantified. Data were compared using a one-way analysis of variance (ANOVA) to determine regional/topographical differences (DMH vs. PFA vs. LH) for HbAA-BERK and HbSS-BERK mice. This analysis was followed by a Kruskal Wallis *post-hoc* test with significant levels set at *p* < 0.05). We used independent *t*-tests to determine whether there were statistical differences between the means of HbAA-BERK and HbSS-BERK mice (activational differences) for: weight, mechanical hyperalgesia, heat hyperalgesia, cold hyperalgesia, grip force and observed immunoreactive cells. All data are represented as mean ± SE, *p* < 0.05.

## Results

Behavioral and immunohistochemical approaches were used to determine pain-related behaviors and investigate whether activational and topographical differences in the subpopulations of orexin in HbAA-BERK and HbSS-BERK mice. We used c-Fos as a marker for neuronal activation in this study. All data reflect observations in female mice since this group expresses higher hyperalgesia than male mice (Kohli et al., 2010; Lei et al., 2016).

### Mechanical Hyperalgesia: Assess Sensitivity to Mechanical Stimulus

**Figure 1:**
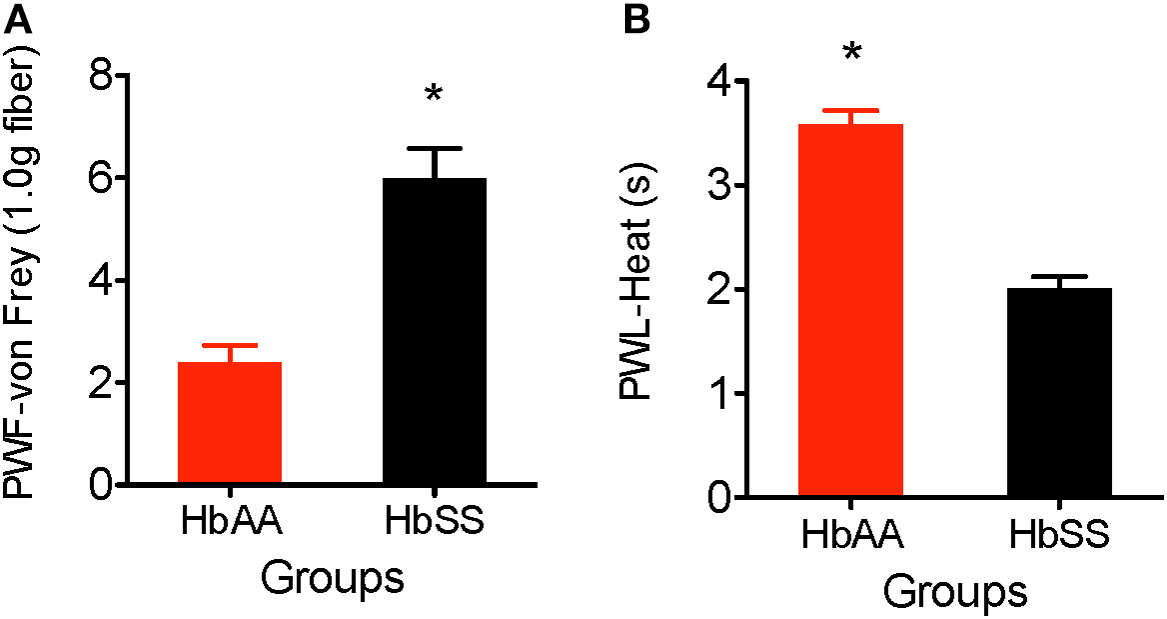
Comparative differences in behavioral assessments for mechanical and heat hyperalgesia in Female HbAA-BERK and HbSS-BERK mice. All data are reflected as mean ± SE, *n* = 9–10/group. **(A)** Mechanical hyperalgesia was measured by paw withdrawal frequency (PWF) in HbAA-BERK and HbSS-BERK mice. HbSS-BERK mice display significantly more PWF than HbAA-BERK mice (^*^p < 0.0001). **(B)** Heat hyperalgesia was measured by paw withdrawal latency (PWL) in response to a heat stimulus in age and sex-matched HbAA-BERK and HbSS-BERK mice. HbAA-BERK mice display significantly greater PWL than HbSS-BERK mice (^*^p < 0.0001).

The von Frey filament (1.0 g, 4.08 mN) was applied for 1–2 s (with enough force to bend the filament) to the plantar surface of each hind paw of HbAA-BERK and HbSS-BERK mice. This stimulus is not characterized as normally painful. However, in animals that have greater tactile sensitivity (HbSS mice), there is a greater response to the filament application. The paw withdrawal frequency evoked when using the von Frey monofilament was significantly higher in HbSS-BERK mice vs. HbAA-BERK control mice (Figure 1A, *p* < 0.0001, 5.99 ± 0.6 vs. 2.4 ± 0.3). This observation in higher paw withdrawal frequency indicated increased hyperalgesia in HbSS-BERK mice.

### Heat Hyperalgesia: Test for Heat Sensitivity

Paw withdrawal latency was measured as the duration of time recorded after the plantar surface of a single hind paw was exposed to a radiant heat stimulus (50 W projector lamp bulb). HbAA-BERK mice display significantly higher paw withdrawal latency vs. HbSS-BERK mice (Figure 1B, *p* < 0.0001, 3.59 ± 0.13 vs. 2.01 ± 0.11). The shorter paw withdrawal latency observed in the HbSS-BERK mice (Figure 1B) indicated increased sensitivity to heat. This heat sensitivity may indicate cutaneous hyperalgesia in HbSS-BERK mice.

### Deep Tissue Hyperalgesia

**Figure 2:**
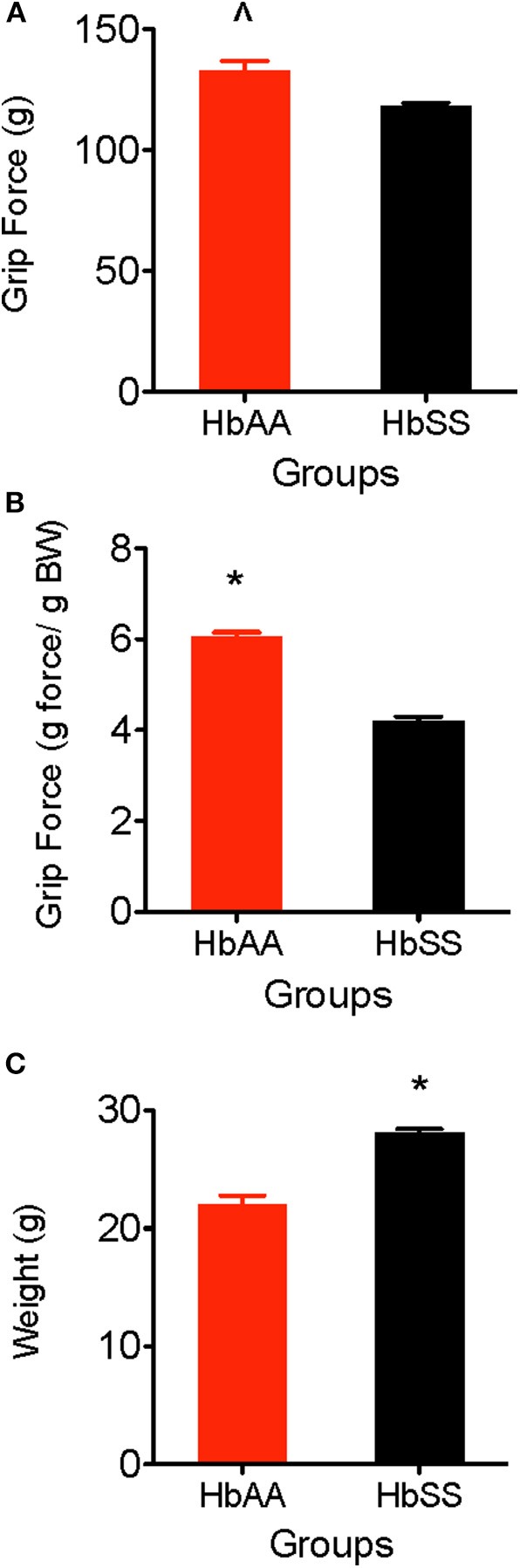
Comparative Differences in deep tissue hyperalgesia and body weight for HbAA-BERK and HbSS-BERK mice. All data are reflected as mean ± SE, *n* = 9–10/group. **(A)** Differences in deep tissue hyperalgesia was assessed by grip force for HbAA-BERK and HbSS-BERK mice. HbAA-BERK mice display significantly more peak forepaw grip strength vs. HbSS-BERK mice (^∧^*p* < 0.005). **(B)** When corrected for weight, HbAA-BERK mice still displayed significantly more grip strength vs. HbSS-BERK mice (^*^p < 0.0001). **(C)** The HbSS-BERK mice in this study were significantly heavier in body weight than HbAA-BERK mice (^*^p < 0.0001).

One of the major consequences of SCD is chronic musculoskeletal pain which can be evidenced by muscle soreness and joint tenderness. Deep tissue hyperalgesia indicates the existence of inherent pain due to activation of visceral, joint, and musculoskeletal nociceptors. In this study, we utilized the grip force test to evaluate musculoskeletal pain in HbAA-BERK and HbSS-BERK mice. Deep tissue hyperalgesia was defined as a decrease in the grip force, which indicates increased nociception. Grip force significantly decreased in HbSS-BERK mice vs. HbAA-BERK control mice (Figure 2A). It was observed that HbAA-BERK mice exerted significantly more grip strength vs. HbSS-BERK mice (Figure 2A, *p* < 0.005, 132.9 ± 3.9 vs. 118.2 ± 1.4, respectively) since a higher force (in g) exerted at the gauge at the time of grip release by the HbAA-BERK mice was recorded.

#### Differences in grip force/body weight

Typically, musculoskeletal strength is greater as weight and muscle development increase. However, it is possible for grip force to not significantly change when expressed per gram body weight if there are underlying physiological factors (i.e., decreased muscle strength, inflammation, increased nociception) that contribute to muscle weakness and pain. When corrected for weight in this study, HbAA-BERK mice still display significantly more grip strength vs. HbSS-BERK mice (Figure 2B, *p* < 0.0001, 6.05 ± 0.10 vs. 4.21 ± 0.09) even though the HbSS-BERK mice were significantly heavier in body weight than the HbAA-BERK mice (Figure 2C, 28.81 ± 0.25 g vs. 22.02 ± 0.77 g, respectively).

### Cold Hyperalgesia: Test for Cold Sensitivity and Behavioral Responses

**Figure 3:**
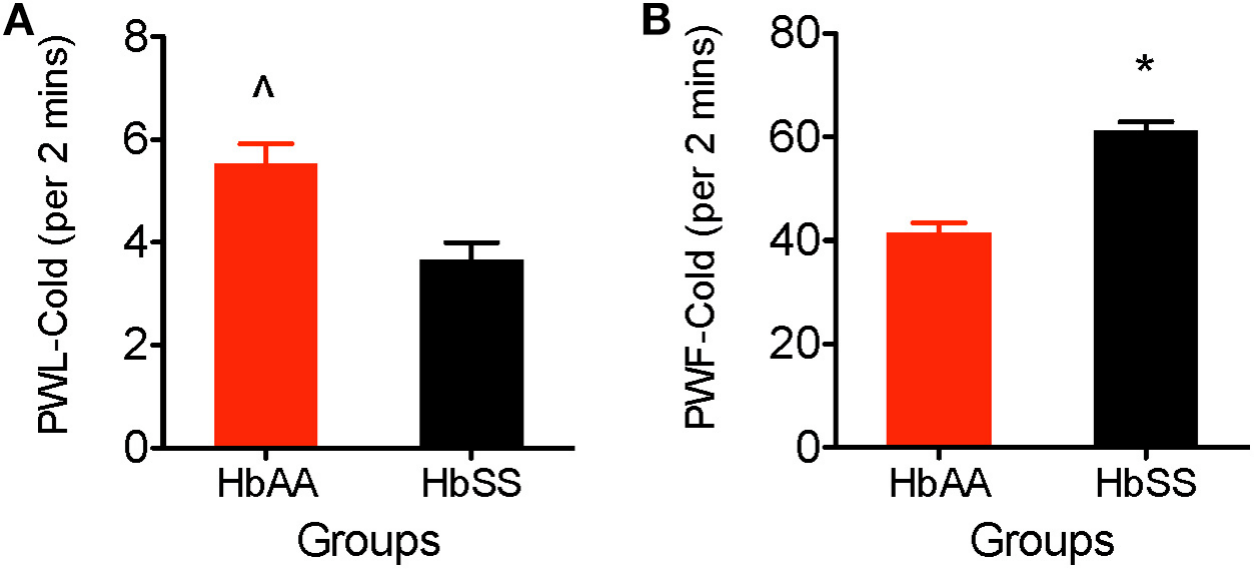
Comparative Differences in behavioral assessments for cold hyperalgesia in female HbAA-BERK and HbSS-BERK mice. All data are reflected as mean ± SE, *n* = 9–10/group. **(A)** HbAA-BERK mice have significantly more PWL vs. HbSS-BERK mice (^∧^*p* < 0.005), therefore, HbSS-BERK mice display more cold hyperalgesia after exposure to a 4°C cold plate, **(B)** HbSS-BERK mice display significantly more behavioral responses to the cold plate temperature of 4°C (^*^p < 0.0001).

We observed a higher sensitivity to the cold stimulus (aluminum plate) in HbSS-BERK vs. HbAA-BERK mice. The HbAA-BERK mice display significantly higher paw withdrawal latency vs. HbSS-BERK mice (Figure 3A, *p* < 0.005, 5.53 ± 0.38 vs. 3.65 ± 0.34). HbAA-BERK mice demonstrated a lower response in lifting either paw and were less likely than HbSS-BERK mice to respond to cold temperatures. HbAA mice spent more time walking around the platform on all four paws before the initial lifting of either paw vs. HbSS-BERK mice. The shorter paw withdrawal latency observed in the HbSS-BERK mice (Figure 3A) indicated increased sensitivity to cold temperature. This cold sensitivity may also indicate cutaneous hyperalgesia in HbSS-BERK mice. In addition, to measuring paw withdrawal latency, behavioral responses due to the exposure to the cold environment were recorded over a 2 min period. Observations were recorded for shivering/body shakes, paw flutter, and consistently lifting paws from the cold plate. HbSS-BERK mice display significantly more paw withdrawal frequency and behavioral responses vs. HbAA-BERK mice (Figure 3B, *p* < 0.0001, 61.18 ± 1.8 vs. 41.53 ± 1.87).

### Quantification for Immunohistochemical Detection of Fos, Orexin, and Orexin-Fos Neurons in the DMH, PFA, and LH

**Figure 4:**
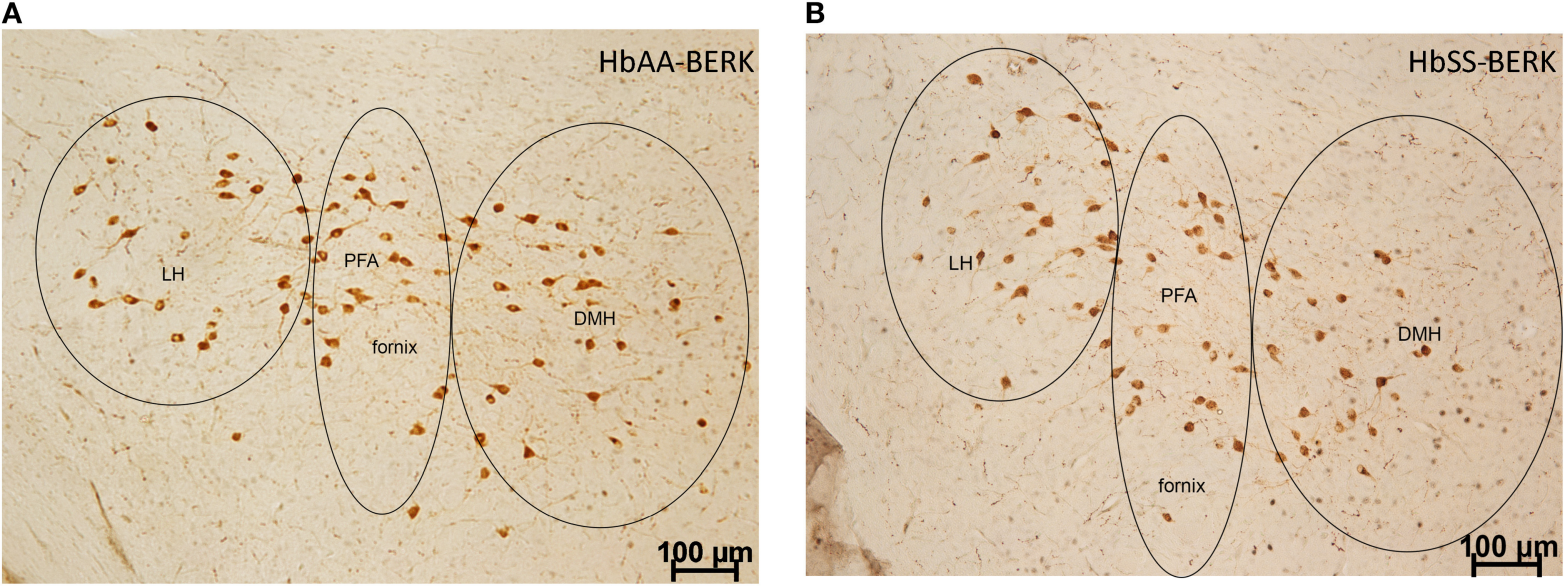
Representation of the pattern of labeling in each hypothalamic subregion. Specific cell types were observed in HbAA-BERK and HbSS-BERK mice following c-Fos and orexin immunohistochemical procedures. **(A)** represents a photo from an HbAA-BERK animal. **(B)** represents a photo from an HbSS-BERK animal. The representative pictures reflect the pattern of labeling for each group and show the three hypothalamic subregions: DMH, PFA, and LH. Objective, 10×.

**Figure 5:**
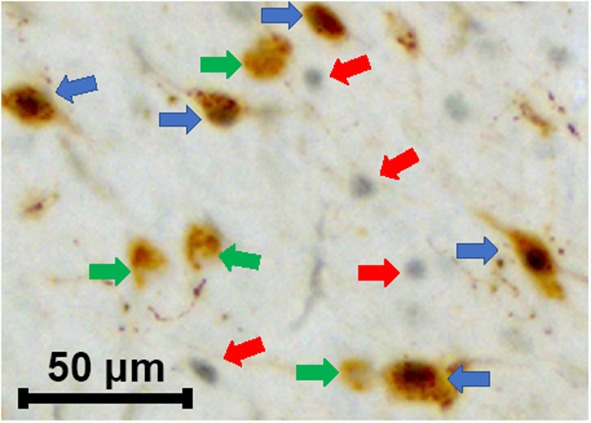
Characterization of various cells types in HbAA-BERK and HbSS-BERK mice after behavioral assessments. The photograph shows the immunoreactivity for single labeled orexin cells (green arrows), single labeled c-Fos (red arrows), and double labeled c-Fos-activated, orexin cells (blue arrows). Black nuclear staining is indicative of c-Fos immunoreactivity and brown cytoplasmic staining is indicative of orexin-A peptide immunoreactivity. Objective, 20×.

To examine whether there were topographical and activational changes in the various subpopulations of orexin neurons located in the DMH, PFA and LH of HbAA-BERK and HbSS-BERK mice, hypothalamic sections were processed for double label immunohistochemistry for c-Fos and orexin-A peptide (Figures 4A,B at −1.94 mm Bregma). There were double labeled neurons (orexin-Fos positive neurons), single labeled orexin neurons, and single labeled c-Fos neurons throughout the DMH, PFA, and LH of both mouse groups (Figure 5).

#### Quantification of Double Labeled Neurons in the LH, PFA, and DMH of HbAA-BERK and HbSS-BERK Mice After Behavioral Tests

**Figure 6:**
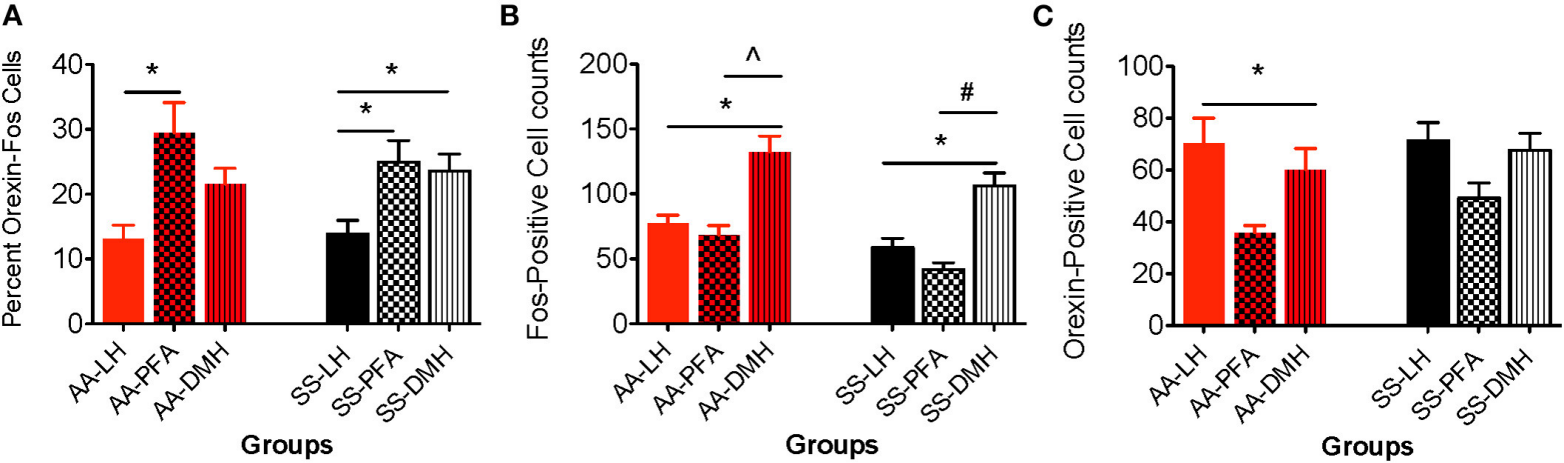
Topographical differences in the immunohistochemical detection and quantification of c-Fos-positive, orexin-positive, and orexin-Fos cells in HbAA- and HbSS-BERK mice after behavioral assessments. All data are reflected as mean ± SE, *n* = 9–10/group. **(A)** The percentage of double labeled cells in the PFA was significantly higher than the percentage of double labeled cells in the LH orexin field of HbAA-BERK and HbSS-BERK mice (^*^p < 0.05). The percentage of double labeled cells in the DMH orexin field was significantly higher than the percentage of double labeled cells in the LH of HbSS-BERK mice (^*^p < 0.05). **(B)** In HbAA-BERK mice, there was a significant difference in the number of single labeled, c-Fos neurons in the LH vs. DMH (^*^p < 0.05). Also, in HbAA-BERK mice, the number of c-Fos cells quantified in the DMH was significantly higher than those observed in the PFA (^∧^*p* < 0.005). In HbSS-BERK mice, there was a significant difference in the number of c-Fos cells in HbSS-BERK mice, when comparing LH vs. DMH (^*^p < 0.05). Additionally, there was a significant difference in the number of c-Fos cells in the PFA vs. DMH (^#^*p* < 0.0005) in HbSS-BERK mice. **(C)** Single-labeled, orexin positive cells were observed in the DMH, PFA, and LH of HbAA and HbSS mice. In HbAA mice, the number of orexin positive neurons was greater in the LH vs. PFA (^*^p < 0.05).

The percentages of orexin-Fos, double labeled neurons were quantified in the LH, PFA and DMH for HbAA-BERK (Figure 6A, 13.2 ± 2.1, 29.4 ± 4.7, 21.6 ± 2.5, respectively) and in the LH, PFA and DMH for HbSS-BERK mice (Figure 6A, 13.9 ± 2.1, 24.94 ± 3.3, 23.6 ± 2.6, respectively). In HbAA-BERK mice, the percentage of orexin-Fos neurons in the PFA was significantly higher than those observed in the LH orexin field (Figure 6A, ^*^p < 0.05). In HbSS-BERK mice, there was a different finding. While the percentage of orexin-Fos neurons was higher in the PFA vs. LH, the percentage of orexin-Fos neurons in the DMH were also significantly higher than those observed in the LH (Figure 6A, ^*^p < 0.05). This difference in topographical activation indicates that a greater number of orexin neurons are recruited/activated in two hypothalamic subregions (DMH and PFA) in the HbSS-BERK mice after behavioral testings.

#### Quantification of Single Labeled Neurons in the DMH, PFA, and LH of HbAA-BERK and HbSS-BERK Mice After Behavioral Tests

Single labeled c-Fos and orexin immunoreactive neurons were observed (Figures 6B,C) and quantified in all three orexin hypothalamic subregions of HbAA-BERK and HbSS-BERK mice.

##### Topographical differences in the number of c-fos neurons between the 3 hypothalamic regions

A one-way ANOVA was used to determine any significant differences in the means of c-Fos neurons quantified in the DMH, PFA and LH regions. In HbAA-BERK mice, there was a significant difference in the number of c-Fos neurons in the LH vs. DMH (77.6 ± 6.1 vs. 132.4 ± 12.2, respectively, ^*^p < 0.05). The number of c-Fos neurons quantified in the DMH was significantly higher than those observed in the PFA (132.4 ± 12.2 vs. 68.2 ± 7.6, *p* < 0.005) in HbAA-BERK mice. *Post-hoc* analysis revealed that there was a significant difference in the number of c-Fos neurons in HbSS-BERK mice, when comparing LH vs. DMH (Figure 6B, 58.5 ± 7.3 vs. 106 ± 10.2, ^*^p < 0.05). Additionally, there was a significant difference in the number of Fos neurons in the PFA vs. DMH (Figure 6B, 41.6 ± 5.3 vs. 106 ± 10.2, respectively, ^#^*p* < 0.0005) in HbSS-BERK mice.

##### Topographical differences in the number of orexin neurons between the 3 hypothalamic regions

A one-way ANOVA was used to determine any significant differences in the means of orexin neurons quantified in the DMH vs. PFA vs. LH regions. In HbAA-BERK mice, there was a significant difference in the number of orexin neurons quantified in the LH vs. PFA (Figure 6C, ^*^p < 0.05). However, there were no other significant differences observed between the regions. There was no significant difference in the number of orexin neurons in HbSS-BERK mice when comparing the three regions Figure 6C (DMH 67.7 ± 6.5, PFA 49.2 ± 5.9, LH 71.3 ± 7.1, *p* = 0.060).

##### Activational differences for c-fos-activated orexin neurons in HbAA-BERK vs. HbSS-BERK mice for each hypothalamic region

**Figure 7:**
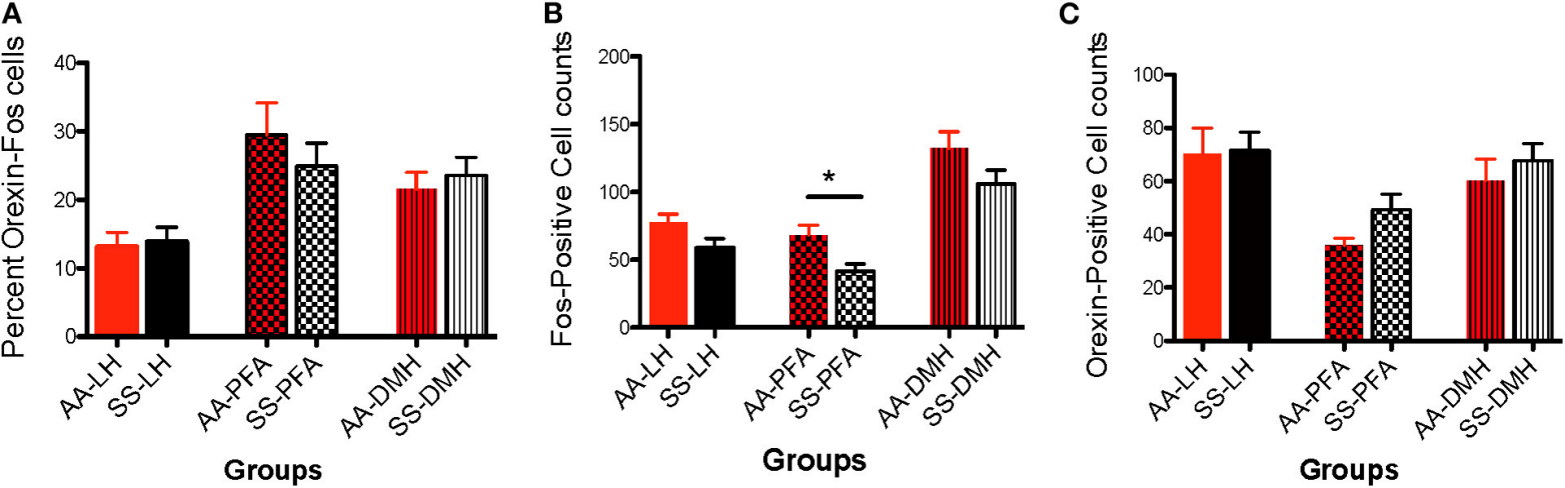
Activational differences in the immunohistochemical detection and quantification of c-Fos-positive, orexin-positive, and orexin-Fos cells in HbAA-BERK and HbSS-BERK mice after behavioral assessments. All data are reflected as mean ± SE, *n* = 9–10/group. **(A)** There was no significant difference in the percentage of double labeled cells in the LH, PFA, and DMH of HbAA-BERK vs. HbSS-BERK mice. **(B)** Single labeled, c-Fos positive cells were observed in the DMH, PFA, and LH of HbAA-BERK and HbSS-BERK mice. HbAA-BERK mice displayed a greater number of c-Fos positive cells in the PFA vs. HbSS-BERK mice (^*^p < 0.05). **(C)** Single-labeled, orexin positive cells were observed in the DMH, PFA, and LH of HbAA-BERK and HbSS-BERK mice, but there was no significant difference in the number of orexin positive cells in HbAA-BERK vs. HbSS-BERK mice.

Data analysis revealed that there was no significant difference in the percentage of LH-located, c-Fos activated orexin neurons from HbAA-BERK vs. HbSS-BERK mice (Figure 7A, 13.2 ± 2.1 vs. 13.9 ± 2.1, *p* = 0.810). There was also no significant difference in the percentage of c-Fos-activated orexin neurons in the PFA from HbAA-BERK vs. HbSS-BERK mice (25.4 ± 2.4 vs. 24.9 ± 3.3, *p* = 0.923). There also was no significant difference in the percentage of DMH-located, c-Fos-activated orexin neurons in HbAA-BERK vs. HbSS-BERK mice (Figure 7A, 21.5 ± 2.5 vs. 23.6 ± 2.6, *p* = 0.575).

##### Activational differences for c-fos neurons in HbAA-BERK vs. HbSS-BERK mice for each hypothalamic region

Data analysis revealed that there was a trend toward significance in the number of c-Fos neurons in the LH from HbAA-BERK vs. HbSS-BERK mice (Figure 7B, 77.6 ± 6.1 vs. 58.5 ± 7.3, *p* = 0.06). There was a significant increase in the number of c-Fos neurons in the PFA in HbAA-BERK vs. HbSS mice (Figure 7B, 68.2 ± 7.6 vs. 41.6 ± 5.3, ^*^p < 0.05). There was no significant difference in the number of DMH-located, c-Fos neurons from HbAA-BERK vs. HbSS-BERK mice (Figure 7B, 132.4 ± 12.2 vs. 106 ± 10.2, *p* = 0.119).

##### Activational differences for orexin neurons in HbAA-BERK vs. HbSS-BERK mice for each hypothalamic region

The presence of single labeled orexin neurons indicated that not all of the orexin neurons within the different subregions were engaged or activated after pain testing (Figure 7C) in HbAA-BERK and HbSS-BERK mice. There was no significant difference in the number of LH-located orexin neurons in HbAA-BERK vs. HbSS-BERK mice (70.3 ± 9.7 vs. 71.3 ± 7.0, *p* = 0.934). This means that the total orexin immunoreactive neuron counts in those subregions were similar in HbAA-BERK vs. HbSS-BERK mice. Similarly, there was no significant difference in the number of PFA-located, orexin neurons from HbAA-BERK vs. HbSS-BERK mice (35.9 ± 2.7 vs. 49.2 ± 5.9, *p* = 0.08). nor a statistical significance in the number of DMH-located, orexin neurons from HbAA-BERK vs. HbSS-BERK mice (Figure 7C, 60.1 ± 8.2 vs. 67.7 ± 6.5, *p* = 0.475).

## Discussion

In the present investigation, we sought to determine whether there were quantitative differences in the activation of orexin neurons after pain testing in a mouse model of SCD. This current study assessed the degree of hyperalgesia expressed in transgenic sickle mice (that express human sickle hemoglobin) vs. control mice (that express normal human hemoglobin) using various pain testing modules and then quantified the immunoreactivity for c-Fos, orexin, and double labeled, c-Fos activated, orexin neurons in the DMH, PFA and LH of these two groups of mice. The behavioral results showed that HbSS-BERK mice display a higher degree of hyperalgesia than HbAA-BERK mice and that while there were no significant activational differences in AA vs. SS mice for the three subregions, topographical differences were observed in HbAA-BERK and HbSS-BERK mice. Overall, the data indicate that the state of the mice (sickle hemoglobin vs. normal hemoglobin) and their sensitivity to painful stimuli may influence activation of orexin neurons within specific hypothalamic subregions.

Our behavioral findings showed that HbSS-BERK mice display significantly greater sensitivity to heat and cold hyperalgesia vs. HbAA mice. The HbSS-BERK mice showed a decreased paw withdrawal latency vs. HbAA mice to the heat stimulus as evidenced by the shorter time interval required to move the forepaw from the floor of the apparatus after being exposed to the heat. Similarly, HbSS-BERK mice displayed decreased paw withdrawal latency vs. HbAA-BERK mice when exposed to the surface of the cold plate. The HbSS-BERK mice lifted their forepaw in a shorter time and displayed a greater number of behavioral responses while exposed to the cold environment. Specifically, there was an increased number of observations for shivering/body shakes, paw flutter, and consistently lifting paws from the cold plate in the HbSS-BERK vs. HbAA-BERK mice. This increase in physical responses to the cold environment indicates that SS mice have more cold sensitivity and may also indicate cutaneous hyperalgesia in HbSS-BERK mice. It is thought that temperature changes and extremes may precipitate painful crises in patients with SCD (Smith et al., 2003) and our observations and others (Lei et al., 2016) support this claim in the HbSS-BERK mice model.

Similarly, HbSS-BERK mice displayed an increase in mechanical hyperalgesia and deep tissue hyperalgesia vs. HbAA-BERK mice with an increased sensitivity to the Von Frey filament and decreased grip force, respectively. The paw withdrawal frequency evoked when using the von Frey monofilament was significantly higher in HbSS mice vs. HbAA-BERK control mice. This observation in higher paw withdrawal frequency indicated increased hyperalgesia in HbSS-BERK mice. The measurement of deep tissue hyperalgesia in the mice was done to model the chronic musculoskeletal pain reported by SCD patients. Information gained from measuring deep tissue hyperalgesia may indicate inherent pain in the mice. Deep tissue hyperalgesia is associated with the activation of visceral, joint, and musculoskeletal pain receptors. The behavioral responses may reflect the muscle and joint tenderness that is often observed during a painful crises.

Our current findings for HbAA-BERK and HbSS-BERK mice during pain testing are consistent with that found in past studies (Kohli et al., 2010; Lei et al., 2016) and support the validity of this model to study neuropathic pain. In agreement with their findings, HbSS mice (with sickle human hemoglobin) display more responses to pain testing indicating increased hyperalgesia vs. HbAA-BERK control mice. Animal models have become increasingly important in understanding neuropathic pain in SCD patient. Transgenic sickle mice that express sickle hemoglobin are one of the best models to date. These mice experience pain episodes similar to those observed in humans. It is more common in females, therefore, we only used female mice in our study. It is estimated that the incidence of neuropathic pain in the SCD population may be twice what is found in other chronic pain populations other than SCD (Brandow et al., 2014). It is believed that neuropathic pain cases occur during painful sickle crises and resolve after the crises ends.

The data from our immunohistochemical studies identified three distinct groups of neurons within the hypothalamic regions of HbSS and HbAA-BERK mice: Fos only-single labeled, orexin only-single labeled, and c-Fos activated, orexin neurons. Differential activation of orexin subpopulations after pain testing in HbSS and HbAA-BERK mice were observed. There was a significant increase in the percentage of double labeled (c-Fos-orexin) neurons in the PFA when compared to those located in the LH of HbSS mice and this same relationship was also observed in HbAA-BERK mice. These patterns in activation of orexin cells reveal subregion, differential activation. This observation has also been reported in the literature for orexin neurons after a myriad of behavioral and pharmacological studies, including those to measure the c-Fos, activation of orexin neurons after behavioral testing for reward, reinstatement, feeding, stress and arousal (Boutrel et al., 2005; Harris et al., 2005; Winsky-Sommerer et al., 2005; Smith et al., 2009; Mahler et al., 2012; Moorman et al., 2017). Our data suggest that hyperalgesia-induced behavioral responses are associated with activation of orexin neurons and highlight anatomically and functionally distinct populations of orexin neurons.

A dichotomy in orexin function was previously proposed (Estabrooke et al., 2001; Harris and Aston-Jones, 2006; Yoshida et al., 2006), indicating that orexin neurons that are located in the DMH and PFA are preferentially associated with homeostasis and arousal/shock. Studies have shown that footshock, restraint or cold-exposure all increase c-Fos immunoreactivity in orexin neurons located in the PFA (Sakamoto et al., 2004; Plaza-Zabala et al., 2010; James et al., 2014). The orexin neurons in the LH were preferentially innervated by brainstem and areas involved in autonomic and visceral processing. These LH-located orexin neurons were activated during reward processing for both food and drugs of abuse and directly correlated with behavioral preference (Harris and Aston-Jones, 2006; Mahler et al., 2014). In another study, orexin neurons in the DMH and PFA were affected by diurnal changes; however the same did not occur for LH orexin neurons (Estabrooke et al., 2001). Additionally, activation of LH orexin neurons correlates with weight gain after the administration of anti-psychotic drugs in male rats, but not in DMH orexin neurons (Fadel et al., 2002).

Our current findings extend this hypothesis by proposing that the association between the orexin system and pain may also affected by functional dichotomy. It is possible that selective topographical activation of specific orexin neuron subpopulations were recruited during hyperalgesia. This may explain the significant differences in the percentage of c-Fos activated orexin neurons in the PFA vs. LH in HbSS and HbAA-BERK mice. Interestingly in HbSS-BERK mice only, the percentage of DMH c-Fos-orexin neurons activated after pain testing was also higher than those observed in the LH. This additional recruitment of activated DMH neurons may be as a result of increased hyperalgesia observed in the HbSS-BERK group. The increased hypersensitivity to the stimuli during the series of pain assessments in HbSS-BERK mice may be due to the afferent and efferent projections to and from the subregions.

Although there was no significant difference in activation of orexin neurons in the HbAA-BERK vs. HbSS-BERK mice (activational differences) nor in the absolute orexin neuron counts, there was a difference in the number of Fos neurons activated in HbAA-BERK vs. HbSS-BERK mice. This last finding was unexpected and may result from the sampling methodology. There was sampling from a subset of sections which may have caused some differences in the c-Fos population counts related to the measured hyperalgesia. However, further studies would need to be conducted to show that these Fos neurons were directly correlated with behavior. In our hands, this increase in Fos was not directly correlated with behaviors after pain testings. Another caveat is that the cell types for the single labeled c-Fos neurons that were recruited or activated after pain testing were not identified in this study. There are a number of other neuropeptides and neurotransmitters in these regions; however, identification of those specific neuronal cell types was beyond the scope of this investigation.

Previous studies have implicated the orexin system in the modulation of pain. In a neuropathic pain model using the partial sciatic nerve ligation in the rat, intrathecal and intracerebroventricular orexin-A administration produced a significant analgesic effect (Yamamoto et al., 2002). In another study, Orexin-A peptide reduced heat evoked hyperalgesia in a rat model of chronic constriction injury of the sciatic nerve, but the same result was not observed with orexin-B peptide (Suyama et al., 2004). This antinociceptive effect from orexin-A may be mediated partly via orexin-1 receptors (OX1R) in the dorsal horn of the spinal cord (Jeong and Holden, 2009; Wardach et al., 2016). Orexin-A produced an analgesic effect mediated by the activation of OX1R using a hot plate test (Bingham et al., 2001). While all of these studies suggest that orexin-A has an analgesic effect on pain, and specifically neuropathic pain, these data do not provide information about the entrained patterns of activation within the subregions. The lateral hypothalamus may facilitate antinociception through spinally descending orexins neurons. It is thought that directly stimulating the lateral hypothalamus produces antinociception mediated by OX1R in the dorsal horn (Wardach et al., 2016). However, it is difficult to interpret these results from (Wardach et al., 2016) as being specific to LH or at least the region that we have categorized as the LH in our current study, since it is possible that stimulation of that hypothalamic region may have also engaged neurons within the PFA and possibly DMH. In past studies, the categorization of orexin subregion boundaries has differed across studies. For this reason, the data from our study are so critical to contributing to understanding the difference in the profiling of orexin neurons. The immunohistochemical data in our study support a distinct sampling of all of the orexin neuron regions. These data provide more understanding and identification for which orexin subpopulations may be involved in pain processing. This is the first published paper to show the topography associated with the activation and/or engagement of the orexin system in a model of hyperalgesia associated with SCD.

In future studies, we seek to elucidate mechanisms to improve the management of neuropathic pain and apply them to develop appropriate interventions. Previous studies have supported the idea that hyperalgesia is reduced by mechanisms that engage spinally descending orexin-A neurons (Wardach et al., 2016). This neuropeptide system offers a novel approach to treat chronic pain and hyperalgesia. In spite of recent evidence for its effect in reducing hyperalgesia in nerve constriction models, there have been no studies to investigate the system as potential target for neuropathic pain specifically in a model of SCD. This current study is the first to show that there is regionally specific activation of orexin neurons as a result of various pain assessments for hyperalgesia (component of neuropathic pain) demonstrated in a mouse model of SCD. We believe that data from these experiments will lay the foundation for a more in-depth investigation for alternative pharmacological therapies to treat neuropathic pain in the SCD population.

In order to develop strategies to treat and even prevent neuropathic pain in SCD, an initial step in this process was to identify whether there were differences in the activation of orexin neurons in sickle mice vs. control mice and to compare the topography of activated orexin neurons. This information provides the knowledge needed to specifically delineate whether specific subpopulations are selectively recruited in sickle mice after pain assessments for hyperalgesia. These findings confirm the activation of the orexin system after pain challenge in sickle mice vs. control mice and provide an initial map for which subpopulations are activated and can be pharmacologically targeted to treat neuropathic pain.

### Final Thoughts

Despite pain being the most common complication of SCD, there is a lack of novel treatments for pain. Advancement in treatment options for neuropathic pain are needed and drugs commonly used to alleviate pain (i.e., opioids, NSAIDs) have not been reliable. The management of neuropathic pain remains challenging because this type of pain does not respond consistently. Although there has been some improvement in treatment options for neuropathic pain research, patients report that their pain is not managed effectively. Opioid compounds have continued to be the primary option to treat pain for several decades. However, chronic opioids use in SCD may adversely affect the peripheral systems, and the development of opioid tolerance or opioid-induced hyperalgesia. There is minimal use of neuropathic pain drugs (gabapentin and hydroxyurea) in the SCD population and this may be due to minimal systemic screening of this type of pain.

A large proportion of SCD patients use opioids to provide limited relief when experiencing chronic pain. However, long term opioid use may produce severe side effects and do not provide a permanent resolution of the pain. Sociocultural factors also provide a barrier to effective pain management in SCD. Ineffective pain assessment and unfounded concerns by health providers regarding addiction have hindered pain management in the SCD population (Brown et al., 2015). The socio-cultural disparity between patients and providers may contribute to the reluctance of health care providers to prescribe narcotics (Shapiro et al., 1997; Elander et al., 2006). More SCD research and changing attitudes concerning care can help to eliminate the barriers that exist. One option to begin to address this disparity is to identify a non-addictive drug that can be used to alleviate pain in the SCD population. We contend that treatments that pharmacologically target the orexin system could be a promising alternative option to reduce pain in SCD and reduce the requirement of opioid analgesics.

## Data Availability Statement

The datasets generated for this study are available on request to the corresponding author.

## Ethics Statement

The animal study was reviewed and approved by the Institutional Animal Care and Use Committee at the University of Minnesota, protocol: KG.

## Author Contributions

KR: planning and conducting experiments, collection of all data, data processing, data analyses and interpretation, figure making, and writing of this manuscript. NS: data processing and writing of this manuscript. HT: conducting experiments and figure making. VA: data analyses. SU: data processing. RT: planning experiments and writing of this manuscript. KG: planning experiments, bred and phenotyped all the mice, interpreting data, and writing of this manuscript.

### Conflict of Interest

The authors declare that the research was conducted in the absence of any commercial or financial relationships that could be construed as a potential conflict of interest. Although there is no conflict with the current work KG reports research grants from Grifols, Cyclerion and 1910 Genetics, outside the submitted work and Honoraria from Novartis, Tautona Group, and CSL Behring.
